# Is Curzerene Responsible for the Bioactive Properties of *Eugenia uniflora*? A Possible Misinterpretation of Bioactive Markers

**DOI:** 10.1021/acsomega.5c08966

**Published:** 2025-11-26

**Authors:** Vinicius Monteiro Schaffka, Raphaela Pereira Guaringue, Larissa Kozan, André Luis Kerek, Cássia Gonçalves Magalhães, Andersson Barison, Barbara Celânia Fiorin

**Affiliations:** † Department of Chemistry, 67883 State University of Ponta Grossa, Av. Carlos Cavalcanti, 4748, 84030-900 Ponta Grossa, Brazil; ‡ Department of Chemistry, 28122 Federal University of Paraná, Av. Coronel Francisco Heráclito dos Santos, 100, 81531-980 Curitiba, Brazil

## Abstract

Essential oils are a complex matrix of volatile compounds produced by many plants of different families with diverse bioactivities. *Eugenia uniflora* (Myrtaceae), popularly known as the pitangueira, is native to Brazil and a source of essential oils, mainly composed of sesquiterpenes such as Furanodiene. Curzerene is the major contributor to most of the bioactive properties assigned to *E. uniflora*. Gas chromatography (GC) is the primary technique for characterizing essential oils. However, sesquiterpenes of the germacrene type may undergo a [3,3]-sigmatropic rearrangement in GC, converting into Elemene-type, leading to these compounds’ misidentification. Curzerene is an Elemene-type compound known to result from the sigmatropic rearrangement of furanodiene. Aiming to demonstrate this evidence, the objective of this study was to perform a thermal treatment of the essential oil from *E*. *uniflora*, to simulate the heating conditions during GC to identify the chemical transformations in this essential oil. Essential oil from *E. uniflora* leaves were obtained by hidrodistillation and was characterized by GC-MS. Nine major sesquiterpenes were identified in the GC-MS analysis of the oil, with the most prevalent being Germacrene B (16.19%), Curzerene (13.28%), and Germacrene D (12.64%). Additionally, β-Elemene and β-Elemenone were identified in lower concentrations. In the NMR analysis, it was possible to identify only four germacrene-type compounds and a small amount of Curzerene. After thermal treatment (240 °C), the Elemenes resulting from the sigmatropic rearrangement were identified. These results suggest that most of the properties assigned to Curzerene throughout the years, which do not combine cold techniques, such as NMR spectroscopy, to characterize the essential oil of *E. uniflora*, were incorrectly assigned and likely belong to furanodiene, evidencing the importance of apply different methods of analysis in some situations.

## Graphical abstract

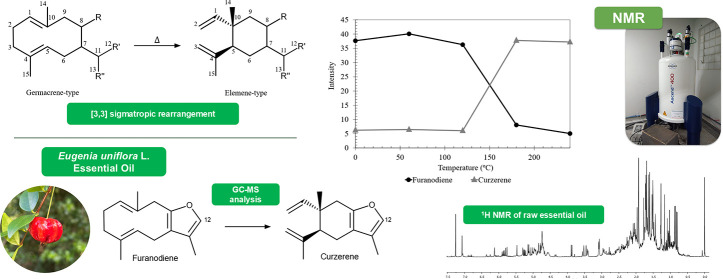

## 1 Introduction

Essential oils are a matrix of volatile and complex compounds characterized by strong odors and significant biological activity. In plants, they play a crucial role in defense against bacteria, viruses, and predators.^1^ These biological properties support the extensive use of essential oils in the food, cosmetics, and pharmaceutical industries.^1,2^ *Eugenia uniflora* L., commonly known as pitangueira, is a native Brazilian tree widely distributed across South America and is well-known for its bioactive essential oil.^3^ Beyond the consumption of its fruits in natura or functional beverages, its leaves are used in infusions, decoctions, and tinctures to treat a variety of diseases, including diarrhea, stomach pain, worm infestations, fever, flu, hyperglycemia, hyperlipidemia, and hypertension.^4,5^ Studies have shown that the composition of secondary metabolites in *E. uniflora* can vary depending on factors such as fruit color, ripening stage,^6^ and seasonal influences.^4,7–10^

The essential oil of *E. uniflora* is predominantly composed of sesquiterpenes, mainly germacrene-type, such as curzerene, germacrene B, germacrone, germacrene D, selina-1,3,7(11)-trien-8-one, and its oxide. Additionally, Elemene-type terpenes are also reported, including furanodiene, β-Elemene, and β-Elemenone.^11–13^

However, those sesquiterpenes are well-documented for their thermal instability. When heat is applied, germacrene-type sesquiterpenes undergo a [3,3]-sigmatropic rearrangement, known as Cope rearrangement, converting into Elemene-type terpenes as presented in Figure 1.^14–16^ This reaction is widely utilized as a synthetic pathway to produce Elemenes from germacrene precursors.^17,18^

**Figure 1:**
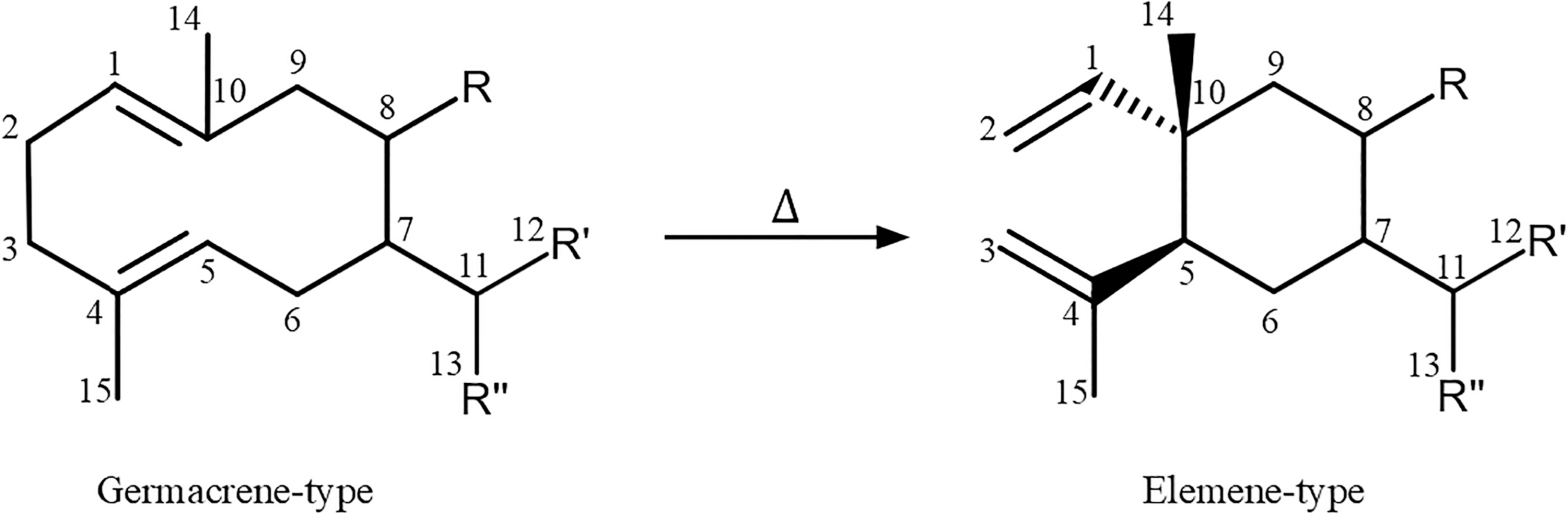
[3,3]-Sigmatropic rearrangement of Germacrene-type into Elemene-type.

The thermal instability of these compounds was first described by Rücker, who identified challenges associated with their determination in essential oils. Later, a method for the quantification of furanodiene using ^13^C NMR spectroscopy was proposed by Baldovini to overcome this limitation. However, the study only reports the conversion of furanodiene into curzerene and lacks comprehensive spectroscopic characterization (^1^H and ^13^C) of these compounds.^19,20^

Gas chromatography (GC) is the main technique for characterizing essential oils, often coupled with mass spectrometry (GC-MS) and the retention index determination is used in the identification of the compounds through comparison with databases.^21^ During GC analysis, essential oils are subjected to multiple heating stages. Initially, the sample is exposed to a high inlet temperature, typically around 240 °C, to ensure complete volatilization. It then enters the oven, where the temperature follows a programmed ramp starting at 60 °C, increasing at 3 °C/min, and reaching up to 240 °C. Finally, the sample encounters the elevated temperatures of the mass spectrometry (MS) detector’s source (250 °C) and interface (280 °C). Even in isocratic methods, where the oven temperature remains constant throughout the analysis, the inlet, source, and interface temperatures are maintained at high levels. Although the sample passes through these stages briefly, the elevated temperatures can induce Cope rearrangements in the essential oil, leading to possible changes in its composition.^22,23^

The accurate characterization of the chemical composition of essential oils is crucial for any study employing them as a biological or analytical matrix, as it supports the interpretation of bioactivity, chemotaxonomic classification, and quality control of samples with economic value added. There are many studies attributing relevant biological activities, classifying specimens, and identifying phenotypic and genetic variations based on the chemical composition of the essential oil of *E. uniflora* L., which is usually characterized by GC-MS.^8,9,11,24^ As an example of the study carried out by de Jesus, found acute anti-inflammatory effect of a rich in curzerene essential oil.^5^ Similarly, Pascoal, reported distinct essential oil composition patterns among *E. uniflora* varieties—yellow, red, and purple fruits—primarily driven by variations in β-Elemene and germacrene D content.^7^ These findings highlight metabolic changes during fruit ripening and underscore the influence of genetic diversity among *E. uniflora* varieties. Such exhibitions are unsatisfactory, because they sustain all their evidence in the characterization of their essential oil only by GC-MS analysis, without considering the Cope rearrangements, a fact that may change the outcome of their research. In this context Nuclear Magnetic Resonance (NMR) spectroscopy is a good alternative for essential oil analysis, since is a robust not destructive technique and able to detect and quantify simultaneously a wide range of metabolites that exceed the concentration of about 5–10 μM.^25^

In order to highlight analytical concerns about the uses of only GC-MS data to access chemical relevance of *E. uniflora* essential oil studies, Nuclear Magnetic Resonance (NMR) spectroscopy was employed to evaluate the chemical composition of the essential oil of *Eugenia uniflora* L. before and after an induced thermal treatment, compared to GC-MS data. The findings provide insights into thermolabile compounds and highlight potential misidentifications of the bioactive properties associated with the sesquiterpenes of *E. uniflora* present in literature.

## 2 Results and Discussion

### 2.1 Characterization of the Essential Oil

Approximately 60 compounds, mostly sesquiterpenes (Germacrene B) and oxygenated sesquiterpenes (Germacrone), were visualized in the essential oil, with the 9 compounds having a relative area above 2%, as shown in Table 1. The identified and most prevalent compounds align with those reported in the literature for *E. uniflora*.^4,11,12^ It is possible to highlight the significant proportion of Germacrene-type sesquiterpenes compared to Elemene-type, except Curzerene, which was found in high concentration.

**Table 1:** Main Components Identified in the GC–MS Analysis of the Essential Oil from Leaves of *E. uniflora*

RRI^a^	RRI^b^	compounds	TIC area (%)
1395	1394	β-Elemene	3.05
1438	1435	γ-Elemene	2.67
1488	1480	Germacrene D	12.64
1503	1500	Curzerene	13.28
1566	1561	Germacrene B	16.19
1605	1605	β-Elemenone	7.68
1702		Furanodiene	4.3
1707	1696	Germacrone	9.03
1755	1755	Oxidoselina-1,3,7(11)-trien-8-one	4.0

a RRI: values of calculated relative retention indices using the column Rtx-5MS (GC–MS) and the *n*-alkanes series C8–C19.

b RRI: published relative retention indices for apolar columns.^21^

The variation in the composition of the essential oil from *E. uniflora* leaves was described in literature,^9^ who classified the specimens into three groups: Group I, containing yellow, dark red, and purple fruits, which showed high concentrations of germacrene B, germacrone, and atractylone; Group II, consisting of light red fruit samples, with high concentrations of curzerene, germacrene D, and germacrene A; and Group III, containing orange-red fruits, with a high content of selina-1,3,7(11)-trien-8-one and oxyselina-1,3,7(11)-trien-8-one. According to this classification, this essential oil fits into Group II.

Chemical transformations of many components found in the *E. uniflora* leaves essential oil result from the heating conditions typically applied during GC–MS analysis, which occur through Cope Rearrangement.^12,19^ Cope rearrangement is a stereospecific [3,3]-sigmatropic rearrangement that proceeds via the most stable chairlike transition state of 1,5-cyclodecadiene, from germacrene-type, to give 1,2-divinylcyclohexane, known as Elemene-type.^14^ Some of the Cope rearrangements found in the *E. uniflora* essential oil are represented in Figure 2.^11,22,23,26^

**Figure 2:**
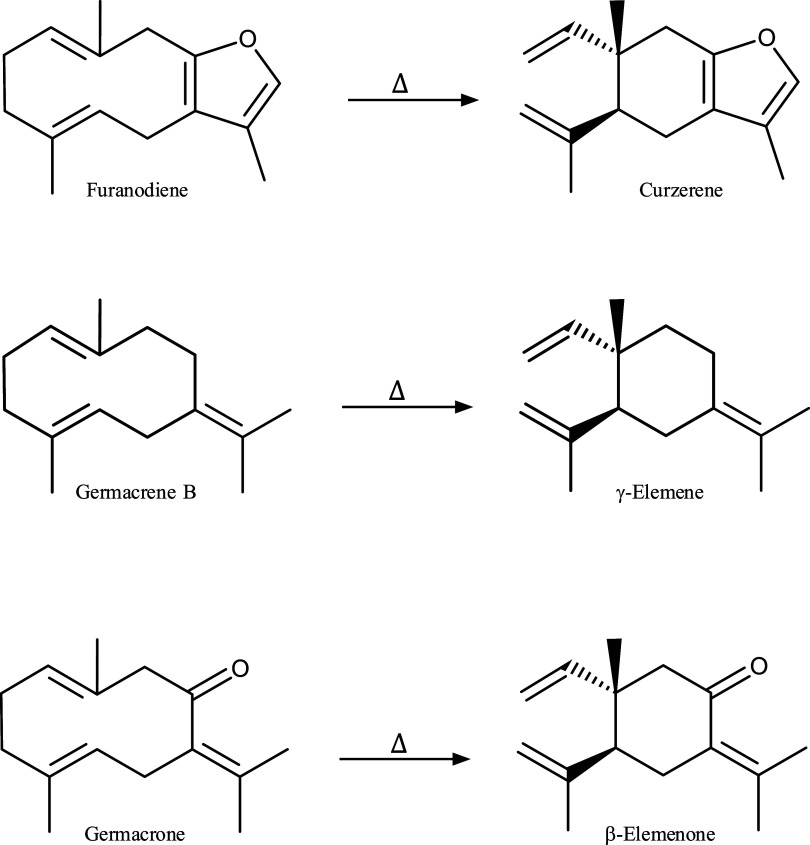
Cope Rearrangements of sesquiterpenes.

The same essential oil was analyzed using NMR spectroscopy to identify its components. Although NMR identification is considerably more labor-intensive than GC-MS analysis, it offers the advantage of being a cold and nondestructive technique. Since the sample does not undergo heating during the process, its stability is preserved throughout the analysis. The conformational differences observed in germacrene-type compounds further increase the spectrum’s complexity, leading to signal broadening in the ^1^H NMR spectra.^27^

A substantial number of signals were observed in the ^1^H spectrum, with the majority concentrated between 0.5 and 3.0 ppm, a region characteristic of terpene compounds. The region between 4.5 and 6.0 ppm also exhibited numerous signals attributed to vinylic protons, observed in sesquiterpenes (Figure 3).

**Figure 3:**
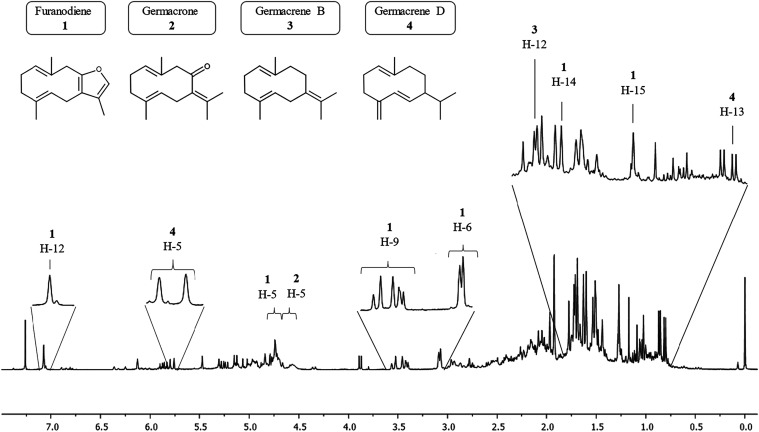
^1^H NMR (400.13 MHz, CDCl_3_) spectra of the raw essential oil.

The assignments of ^1^H and ^13^C signals were carried out using HSQC, selective HSQC, and HMBC spectra (Supporting Information). These assignments were further corroborated by comparing the obtained results with spectral data available in the literature.^17,28–30^

Unlike GC-MS, the NMR analysis has a limited sensitivity for compounds with very low concentrations. Therefore, only the most prevalent compounds could be identified. The compounds identified were Germacrene D, Germacrene B, Furanodiene, and Germacrone, predominantly germacrene-type compounds. The assigned signals are Germacrone NMR ^1^H (CDCl_3_, 400 MHz) δH (multiplicity; *J* in Hz): 1.44 (sl), 1.62 (s), 1.72 (s), 1.77 (s), 2.10–2.15 (m), 2.38–2.40 (m; Ha and Hb), 2.93–2.87 (m), 2.94–3.42 (m), 4.72 (m), 4.98 (m). NMR ^13^C (CDCl_3_, 100 MHz) δC: 15.62 (C-15), 16.73 (C-14), 19.92 (C-12), 22.41 (C-13), 24.1 (C-2), 29.25 (C-6), 38.18 (C-3), 56.01 (C-9), 125.5 (C-5), 126,7 (C-10), 129.5 (C-7), 132.6 (C-1), 135.1 (C-4), 137.3 (C-11), 207.9 (C-8); Germacrene B NMR ^1^H (CDCl_3_, 400 MHz) δH (multiplicity; *J* in Hz): 1.49 (s), 1.53 (s), 1.69 (s), 1.71 (s), 1.83 (m), 1.95–2.15 (m), 2.29–2.04 (m), 2.16 (m), 2.54 (m), 4.56 (m), 4.72 (m). NMR ^13^C (CDCl_3_, 100 MHz) δC: 16.23 (C-15), 16.24 (C-14), 20.43 (C-12), 20.80 (C-13), 25.78 (C-2), 32.54 (C-8), 32.54 (C-9), 38.89 (C-3), 38.89 (C-6), 126.35 (C-7), 126.90 (C-1), 128.20 (C-5), 131.66 (C-4), 133.56 (C-11), 137.13 (C-10); Germacrene D NMR ^1^H (CDCl_3_, 400 MHz) δH (multiplicity; *J* in Hz): 0.80 (d; 6.83), 0.86 (d; 6.83), 1.43 (m), 1.51 (sl), 2.01 (m), 4.9–4.74 (m), 5.12 (m), 5.26 (d; 15.88), 5.77 (d;15.88). NMR ^13^C (CDCl_3_, 100 MHz) δC: 15.91 (C-14), 19.34 (C-12), 20.78 (C-13), 52.77 (C-11), 52.96 (C-7), 109.10 (C-15), 129.26 (C-1), 133.68 (C-6), 134.00 (C-10), 135.61 (C-5), 148.89 (C-4); Furanodiene NMR ^1^H (CDCl_3_, 400 MHz) δH (multiplicity; *J* in Hz): 1.27 (sl), 1.60 (sl), 1.79–2.24 (m), 1.92 (d; 1.21), 2.10–2.14 (m), 3.08 (m), 3.43 (d; 15.71), 3.54 (d; 15.71), 4.74 (m), 4.93 (m), 7.07 (q; 1.21 and 2.38). NMR ^13^C (CDCl_3_, 100 MHz) δC: 8.89 (C-13), 16.23 (C-15), 16.49 (C-14), 24.47 (C-6), 26.80 (C-2), 39.51 (C-3), 40.92 (C-9), 118.86 (C-7), 121.93 (C-11), 127.59 (C-5), 128.82 (C-4), 129.03 (C-1), 134.36 (C-10), 135.98 (C-12), 149.76 (C-8).

Only a few signals related to Curzerene were detected regarding Elemene-type compounds, such as the signal at δ 7.07 ppm (H-12, q; *J* = 1.21 Hz, 2.38 Hz), indicating its low concentration. No signals related to β-Elemene, γ-Elemene, oxyselina-1,3,7(11)-trien-8-one, or β-Elemenone were found.

Considering that the compounds not clearly identified by NMR analysis are products of the Cope rearrangement, the discrepancy observed in the characterization of the essential oil can be attributed primarily to the thermal instability of the *Eugenia uniflora* essential oil.^20,23,26^

Thus, GC-MS characterization does not accurately reflect the accurate composition of the essential oil, but rather represents a sequence of thermal degradation products, which are detected in significant quantities only after their formation during the chromatographic process, as curzerene determination shows. The thermal treatment applied to the essential oil supports this conclusion.

### 2.2 Thermal Treatment of the Essential Oil

To confirm the observations, the same essential oil was subjected to thermal treatment using a melting point apparatus to ensure precise temperature control. The thermal treatment was designed to replicate the temperature ramp experienced by the sample during GC-MS analysis, with aliquots collected at 60, 120, 180, and 240 °C. The aliquots were subjected to ^1^H and ^13^C NMR analysis for compound identification and visualization of thermal rearrangements (Figure 4).

**Figure 4:**
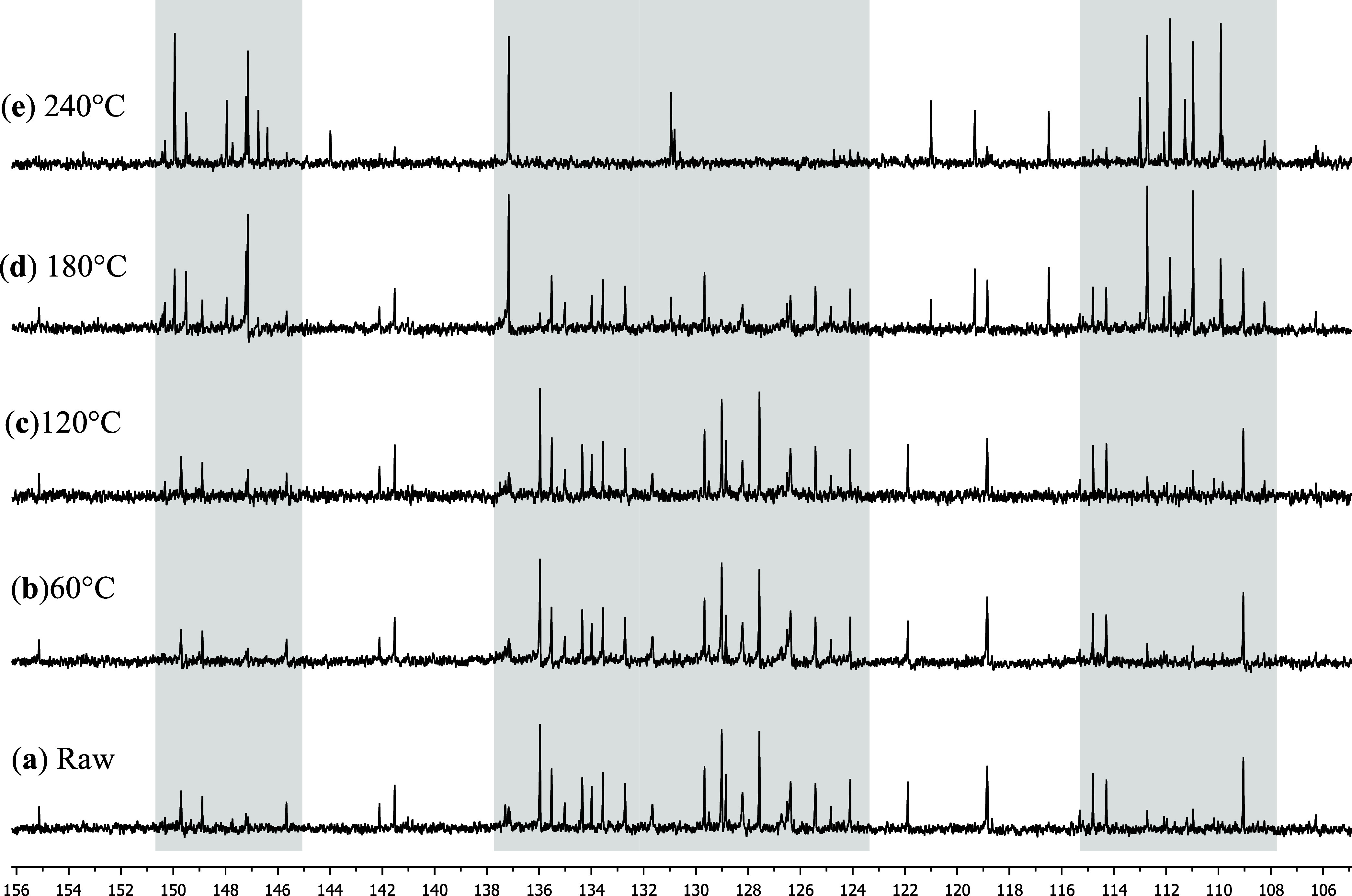
^13^C NMR (100.13 MHz, CDCl_3_) spectra of the thermal treated essential oil of *E. uniflora.* (a) Raw essential oil, (b) 60 °C, (c) 120 °C, (d) 180 °C and (e) 240 °C.

It was observed that, up to the aliquot treated at 120 °C, no significant changes occurred, as seen in the ^13^C spectrum, indicating that up to this temperature, there is insufficient energy in the system to promote the complete conversion of these compounds. This result aligns with the literature, which establishes that the most stable and low-energy conformation for these compounds is of the germacrene type (1,5-cyclodecadiene), and that additional energy is required to drive such reactions.^15,18^ This is exemplified in the work undertaken by Faraldos, where the complete conversion of Germacrene A into β-Elemene was achieved by heating a solution of (+)-germacrene A in toluene at reflux.^27^

In the spectrum obtained at 180 °C, it is evident that the thermal rearrangements begin to occur, with some signals corresponding to the Elemene-type emerging as the intensity of the signals associated with the germacrene decreases. As can be seen in the regions between 108–115 ppm, corresponding to the terminal vinylic carbons of Elemene-type compounds increasing, the region between 123–128 ppm assigned to the vinylic carbons of germacrene-type compounds decreasing, and 146–151 ppm representing the region of the furan ring carbons present in curzerene and furanodiene, changing their chemical sifts (Figure 4d). At 240 °C (Figure 4e), most of the reagent’s signals disappear, leaving only the signals of their thermal degradation product. Several compounds which were identified in the GC-MS analysis, but could not be detected in the raw sample by NMR spectroscopy, were successfully identified in the spectrum at 240 °C, including Curzerene, γ-Elemene, and β-elemonone (Figure 5).

**Figure 5:**
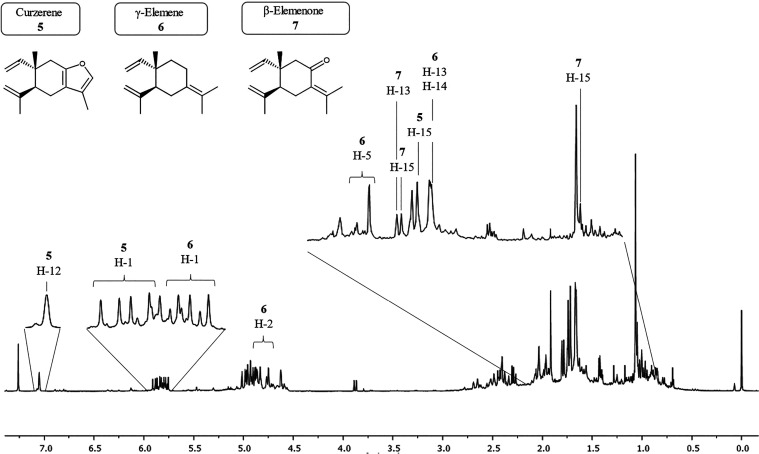
^1^H NMR (400.13 MHz, CDCl_3_) spectra of the 240 °C essential oil.

The assigned signals are Curzerene NMR ^1^H (CDCl_3_, 400 MHz) δ_H_ (multiplicity; *J* in Hz): 1.06 (s), 1.72 (sl), 1.91 (d; 1.05), 2.29 (t; 7.37), 2.35 (dl; 16.49), 2.41 (m), 2.67 (dl; 16.49), 4.88 (m), 4.96 (m), 5.87 (dd; 17.58 and 10.70), 7.05 (q; 1.05 and 2.26). NMR ^13^C (CDCl_3_, 100 MHz) δ_C_: 8.13 (C-13), 19.54 (C-14), 24.19 (C-6), 24.46 (C-15), 36.09 (C-9), 40.11 (C-10), 50.00 (C-5), 109.92 (C-3), 110.97 (C-2), 116.50 (C-7), 119.35 (C-11), 137.18 (C-12), 147.21 (C-4), 147.75 (C-1), 149.50 (C-8); γ-Elemene NMR ^1^H (CDCl_3_, 400 MHz) δ_H_ (multiplicity; *J* in Hz): 1.06 (s), 1.66 (sl), 1.66 (sl), 1.74 (sl), 1.95 (m), 4.83 (m), 4.86 (m), 5.79 (dd; 17.62 and 10.72). NMR ^13^C (CDCl_3_, 100 MHz) δ_C_: 16.81 (C-14), 19.92 (C-12), 20.04 (C-13), 24.80 (C-15), 39.94 (C-10), 53.03 (C-5), 111.87 (C-3), 112.71 (C-2), 121.00 (C-11), 130.94 (C-7), 147.21 (C-4), 149.95 (C-1); β-Elemenone NMR ^1^H (CDCl_3_, 400 MHz) δ_H_ (multiplicity; *J* in Hz): 1.05 (s), 1.78 (sl), 1.80 (sl), 2.03 (sl), 2.28 (d; 15.36), 2.38 (m), 2.46 (d; 15.36), 2.53 (m), 2.64 (m), 4.91 (m), 4.92 (m), 5.80 (dd; 17.48 and 10.80). NMR ^13^C (CDCl_3_, 100 MHz) δ_C_: 19.17 (C-14), 22.57 (C-13), 23.34 (C-12), 24.81 (C-15), 32.00 (C-6), 41.90 (C-10), 50.67 (C-5), 54.01 (C-9), 112.12 (C-2), 113.00 (C-3), 130.99 (C-7), 143.99 (C-11), 146.70 (C-4), 146.72 (C-1), 202.71 (C-8).

When the HSQC spectrum in the 100–130 ppm region between the raw sample and at 240 °C was analyzed, it was observed that, despite the broadening of the vinyl hydrogen signals, the ^1^H–^13^C single bound couplings of Elemene-type compounds occur between low field terminal carbons, in the range of 109–115 ppm, while the vinyl hydrogens of germacrene-type compounds exhibit couplings with nonterminal carbons, distributed in high field frequencies, between 125–130 ppm (Figure 6).

**Figure 6:**
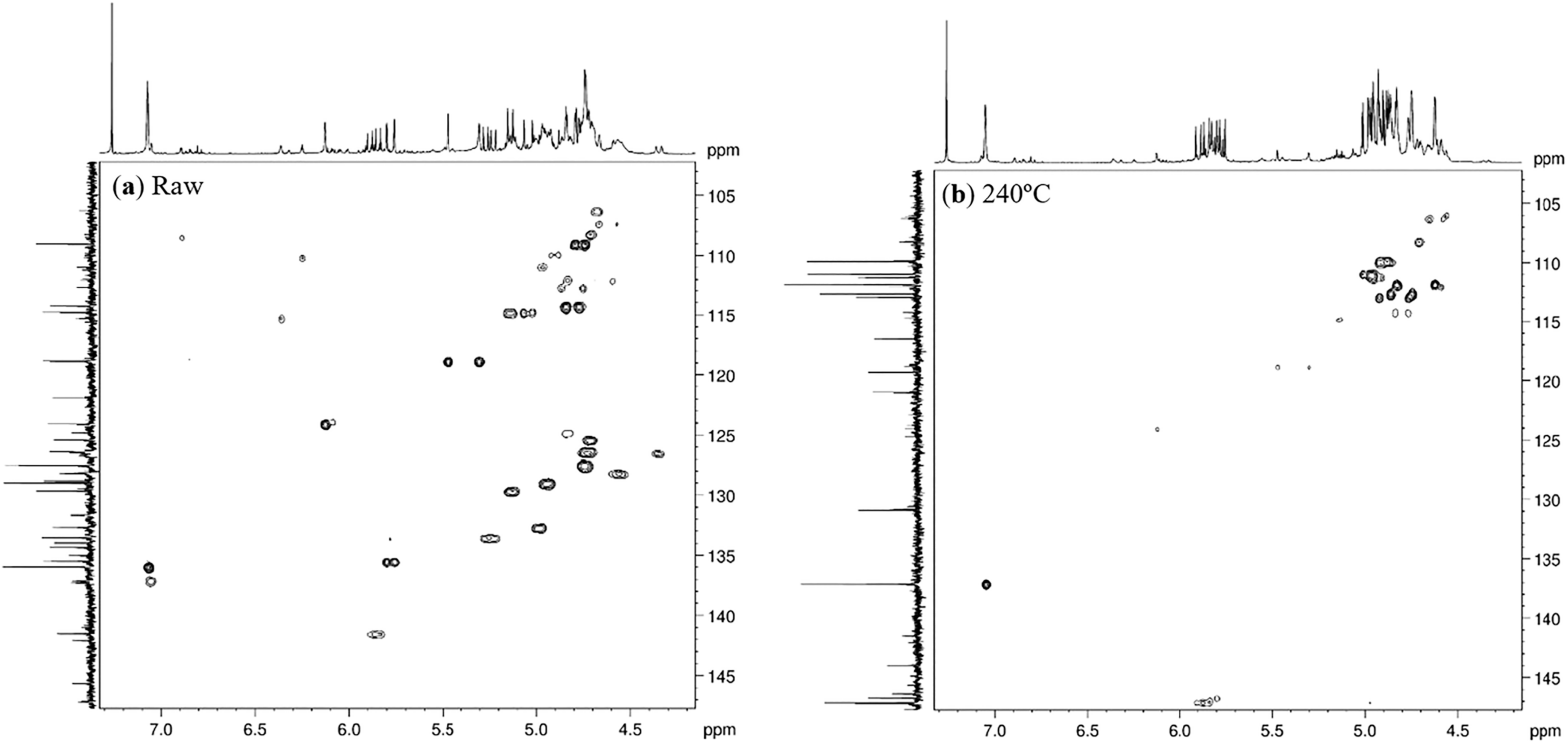
^1^H–^13^C HSQC NMR spectra of (a) raw essential oil and (b) of 240 °C.

Several studies utilize the results of GC-MS analysis to claim that the percentage variation between germacrene-type and Elemene-type compounds can be used as a classification criterion to confirm a specific approach.^5,8–10,13,31^ An example of this is the study carried out by Costa, in which the influence of the fruit biotypes on the chemical composition and antifungal activity *of E. uniflora* essential oils was evaluated.^8^ In their multivariate statistical analysis, they found that the percentage of TIC (total ion chromatogram) related to β-Elemene and curzerene was sufficient to categorize a distinct group compared to the one showing higher proportions of Germacrene B and Germacrone. A similar approach was used by Raupp, when comparing the yield and composition of *E. uniflora* essential oil according to seasonality, significant differences in the percentages of curzerene, germacrene B, and germacrone were found.^31^ Such approaches, however, have failed to address their purposes mainly due to the imprecise determination of the chemical composition of their essential oil. The determination of any observed causality regarding the composition of specimens under different conditions must always be accompanied by a precise characterization of the study matrix.

Curzerene stands out as the most extensively described compound in the essential oil of *E. uniflora* in terms of its biological properties, emphasizing the importance of its accurate characterization.^2,4,5^ This metabolite is an effective and selective antileishmanial agent^32^ It was demonstrated that inhibition of GSTA4 by curzerene correlates with positive outcomes in glioma models, and thus, this molecule is a candidate drug for the treatment of glioma.^33^ The high proportion of curzerene indicated by GC-MS analysis was observed only through ^1^H NMR in samples heated to 240 °C. By integrating the H-12 signals of furanodiene and curzerene and plotting against temperature, it was found that, in the nonheated sample, furanodiene is present in significantly higher proportions than curzerene. Above 150 °C, the conversion of furanodiene into curzerene was detected, with a substantially higher proportion relative to furanodiene at 240 °C (Figure 7).

**Figure 7:**
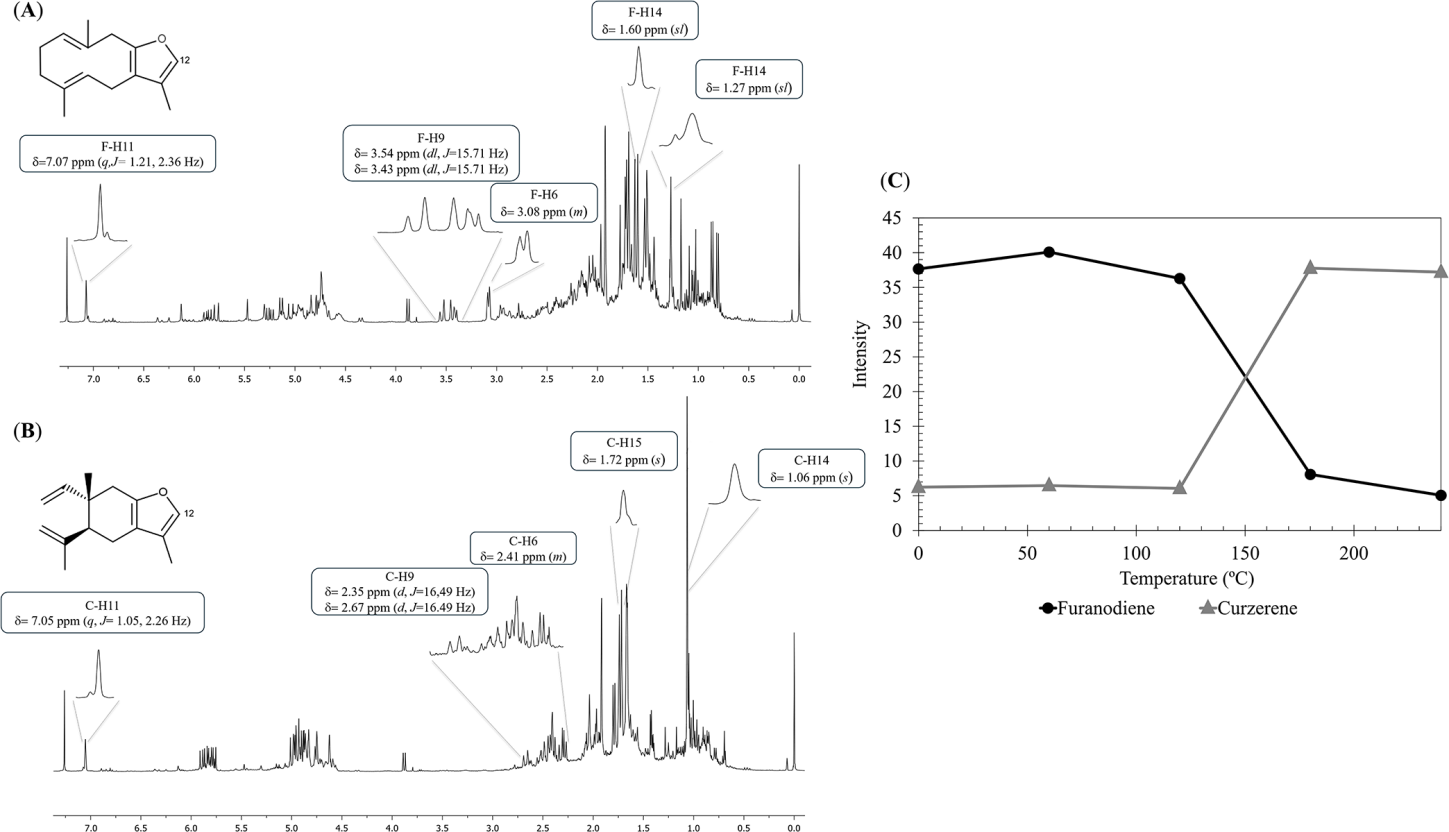
Thermal rearrangement of furanodiene into curzerene. (A) ^1^H NMR spectrum highlighting the signals of furanodiene. (B) ^1^H NMR spectrum highlighting the signals of curzerene. (C) Variation in the intensity of the H-12 signal with temperature, demonstrating the conversion of furanodiene (●) into curzerene (▲).

If the results were based solely on GC-MS analysis and a relevant property was attributed to this essential oil due to the high percentage of curzerene, this property could be ascribed to a thermal degradation product of furanodiene. When employing a cold technique, it becomes evident that only furanodiene is present in high proportions, contrasting once again with chromatographic analysis. It is possible that most of the properties assigned to Curzerene throughout the years, which do not combine cold techniques, such as NMR spectroscopy, to characterize the essential oil of *E. uniflora*, were incorrectly assigned and likely belong to furanodiene.

Although the results indicate a significant analytical limitation in essential oil analysis, it should be emphasized that the chemical composition of a specimen is unique and easily influenced by genotypic and phenotypic factors. Therefore, a single analysis of one essential oil does not fully represent the chemical behavior of the species in its entirety. Cope rearrangements are well-known, and some studies have already detected them in *E. uniflora* essential oils.^11,34^ Despite being reported in the literature since 1977 and reinforced in 2001 by Baldovani, numerous studies continue to rely solely on chemical characterization by GC-MS, demonstrating the need for work with impact in analytical chemistry applied to natural products. The insights presented herein challenge established bioactivity attributions, provide high-quality NMR data, and underscore the need for methodological rigor in essential oil research. In the era of “omics” technology in natural products studies, the complementary aspects of mass spectrometry (MS)- and nuclear magnetic resonance (NMR)-based techniques must be taken into consideration.^35^

## 3 Conclusions

The investigation of the composition of *E. uniflora* essential oil by NMR and the thermal treatment performed proved that curzerene may come from furanodiene due to the transformations that occur in the matrix during gas chromatographic analysis. These findings have significant implications for the investigation into the chemical composition of *E. uniflora* essential oil and the evaluation of the biological properties of its sesquiterpenes. Since each technique has advantages and disadvantages, combining GC-MS analysis with a cold technique, such as NMR is important to guarantee the proper identification of the compound genuinely responsible for any property assigned to *E. uniflora* essential oil.

## 4 Materials and Methods

### 4.1 Plant material and Essential Oil

*Eugenia uniflora* L. leaves were collected at the State University of Ponta Grossa (PR, Brazil) in June 2022. The average temperature during collection was 21 °C, with 60% humidity and no recorded precipitation in the previous 24 h. (25°05′42.2″S 50°06′07.3″W) The specimen was deposited in the herbarium of Universidade Estadual de Ponta Grossa (HUPG), under deposit number HUPG-22452. Genetic patrimony access and traditional knowledge procedures were completed, and the project was registered in SisGen (A23FAEE). The leaves were air-dried at room temperature, shielded from light (120 g), ground, and subjected to hydrodistillation in a Clevenger apparatus for 2.5 h. The essential oil was collected in diethyl ether, which was then evaporated to isolate the oil. The oil was subsequently dried with anhydrous sodium sulfate (Na_2_SO_4_) to remove any residual moisture and stored under refrigeration to preserve its chemical integrity for further analysis.

### 4.2 GC-MS Analysis

The gas chromatographic analyses of the essential oils (10 mg mL^–1^) were performed using a gas chromatography–mass spectrometry system (Shimadzu GCMS-QP2020 Gas Chromatograph). The analyses were conducted on an RTx-5MS capillary column (30 m × 0.25 mm internal diameter × 0.25 μm film thickness), and the analytical conditions were as follows: split ratio of 1/10, injector temperature at 250 °C, ion source at 250 °C, and interface at 280 °C. The temperature program of the oven was set at 60 °C for 5 min, followed by a temperature ramp of 3 °C/min until reaching the final temperature of 240 °C. The components were identified based on the relative retention index, calculated for each constituent by injecting a series of *n*-alkane standards (C8–C20) under the same sample conditions and comparing them with tabulated values as well as by comparing the obtained mass spectra with the mass spectra database and literature comparisons.^21^

### 4.3 Temperature Ramp

The thermal treatment of *Eugenia uniflora* L. essential oil was performed to simulate the GC-MS temperature ramp. A melting point apparatus was used to control the temperature precisely during the heating process. Approximately 1.5 mL of the essential oil was placed in a glass vial and then subjected to a controlled heating rate of 3 °C/min. The temperature was progressively increased, and aliquots of the oil were collected (30 μL) at specific temperatures: 60, 120, 180, and 240 °C. These samples were subsequently analyzed by NMR to assess the effects of thermal treatment on the oil’s composition.

### 4.4 NMR Analysis

All NMR spectra were acquired at 298 K on a Bruker AVANCE III spectrometer operating at 9.4 T, observing ^1^H at 400.13 MHz and ^13^C at 100.13 MHz, equipped with a broadband probe. The essential oil was solubilized in CDCl_3_ with 0.05% of tetramethylsilane (TMS) used as an internal reference. The ^1^H NMR spectra were recorded utilizing 65k time-domain data points across a spectral width of 20.00 ppm. This setup provided a digital resolution of 0.24 Hz. The data were obtained using a single 30° excitation pulse, with a relaxation delay of 1.0 s, and an average of 128 scans. The ^13^C NMR spectra was carried out using the *zgpg30* pulse sequence utilizing 64k time-domain data points across a spectral width of 238 ppm, with an acquisition time of 1.3631 s, providing a digital resolution of 0.7434 Hz and a relaxation delay of 2.0s and an average of 2k scans.

The HSQC and HMBC experiments were conducted using standard pulse sequences from the Bruker library, with modifications to the number of acquired points to improve resolution, 2048 in F2 and 512 in F1.

## Supplementary Material



## Notes

Figure S1: ^1^H NMR (400.13 MHz, CDCl_3_) spectrum of the raw essential oil; Figure S2: ^13^C NMR (100.13 MHz, CDCl_3_) spectrum of the raw essential oil; Figure S3: Selective ^1^H–^13^C HSQC of the raw essential oil; Figure S4: ^1^H–^13^C HSQC of the raw essential oil; Figure S5: ^1^H–^13^C HMBC of the raw essential oil; Figure S6: ^1^H NMR (400.13 MHz, CDCl_3_) spectrum of the 60 °C essential oil; Figure S7: ^13^C NMR (100.13 MHz, CDCl_3_) spectrum of the 60 °C essential oil; Figure S8: ^1^H NMR (400.13 MHz, CDCl_3_) spectrum of the 120 °C essential oil; Figure S9: ^13^C NMR (100.13 MHz, CDCl_3_) spectrum of the 120 °C essential oil; Figure S10: ^1^H NMR (400.13 MHz, CDCl_3_) spectrum of the 180 °C essential oil; Figure S11: ^13^C NMR (100.13 MHz, CDCl_3_) spectrum of the 180 °C essential oil; Figure S12: Selective ^1^H–^13^C HSQC of the 180 °C essential oil; Figure S13: ^1^H–^13^C HSQC of the 180 °C essential oil; Figure S14: ^1^H–^13^C HMBC of the 180 °C essential oil; Figure S15: ^1^H NMR (400.13 MHz, CDCl_3_) spectrum of the 240 °C essential oil; Figure S16: ^13^C NMR (100.13 MHz, CDCl_3_) spectrum of the 240 °C essential oil; Figure S17: Selective ^1^H–^13^C HSQC of the 240 °C essential oil; Figure S18: ^1^H–^13^C HSQC of the 240 °C essential oil; Figure S19: ^1^H–^13^C HMBC of the 240 °C essential oil; Table S1: GC-MS analysis of *E. uniflora* essential oil. Figure S 20: GC-MS chromatogram of the *Eugenia uniflora* L. essential oil (PDF)
