# Supplementary material for: Is Curzerene Responsible for the Bioactive Properties of Eugenia uniflora? A Possible Misinterpretation of Bioactive Markers
Source: ACS Omega. 2025 Nov 26;10(48):59425–34. doi: 10.1021/acsomega.5c08966 (PMC12771166; doi:10.1021/acsomega.5c08966)
Supplement: Supplementary file 1 [file ao5c08966_si_001.pdf]

Supporting Information

Is Curzerene responsible for the bioactive properties of *Eugenia uniflora*? A possible misinterpretation of bioactive markers.

Vinicius Monteiro Schaffka[a], Raphaela Pereira Guaringue[a], Larissa Kozan[a], André Luis Kerek[a], Cássia Gonçalves Magalhães[a], Andersson Barison[b], Barbara Celânia Fiorin[a]*

[a]*Department of Chemistry, State University of Ponta Grossa, Av. Carlos Cavalcanti, 4748, 84030-900, Ponta Grossa, Brazil.*

[b]*Department of Chemistry, Federal University of Paraná, Av. Coronel Francisco Heráclito dos Santos, 100, 81531-980, Curitiba, Brazil.*

bcfiorin@uepg.br*

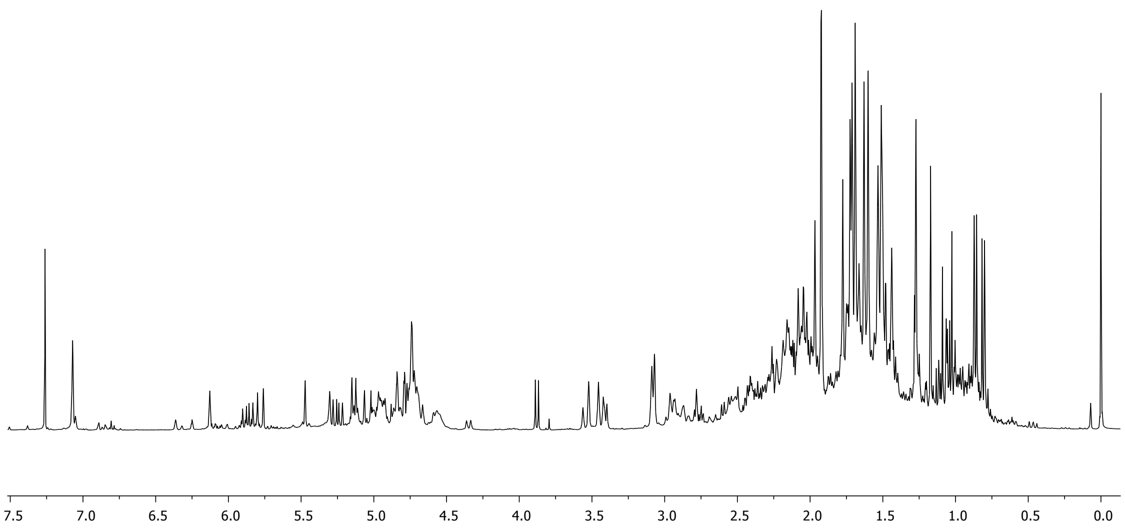

Figure S 1: ^{1}H NMR (400.13 Mhz, $CDCl_3$) spectra of the raw essential oil.

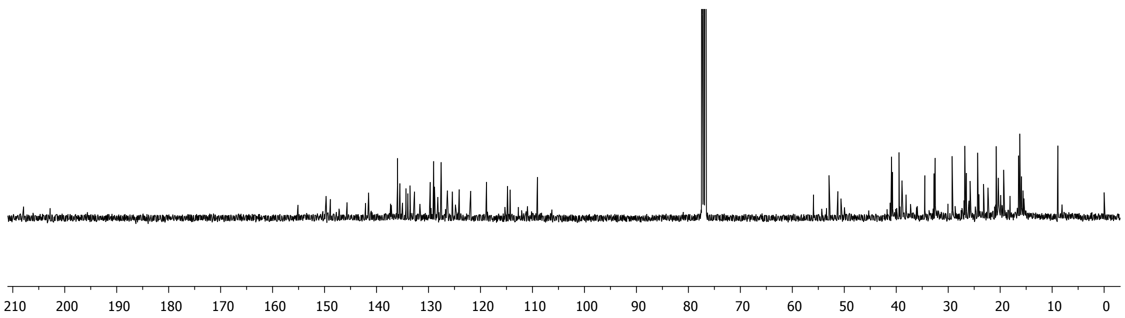

Figure S 2: ^{13}C NMR (100.13 Mhz, $CDCl_3$) spectra of the raw essential oil.

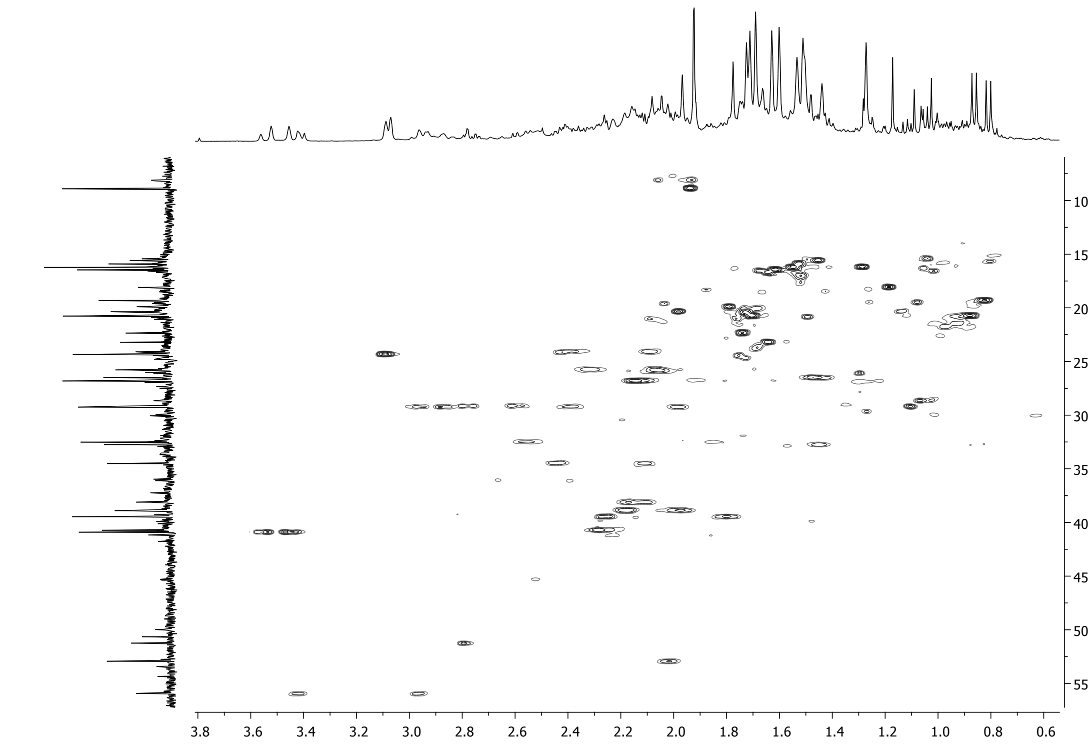

Figure S 3: Selective ^{1}H-^{13}C HSQC of the raw essential oil.

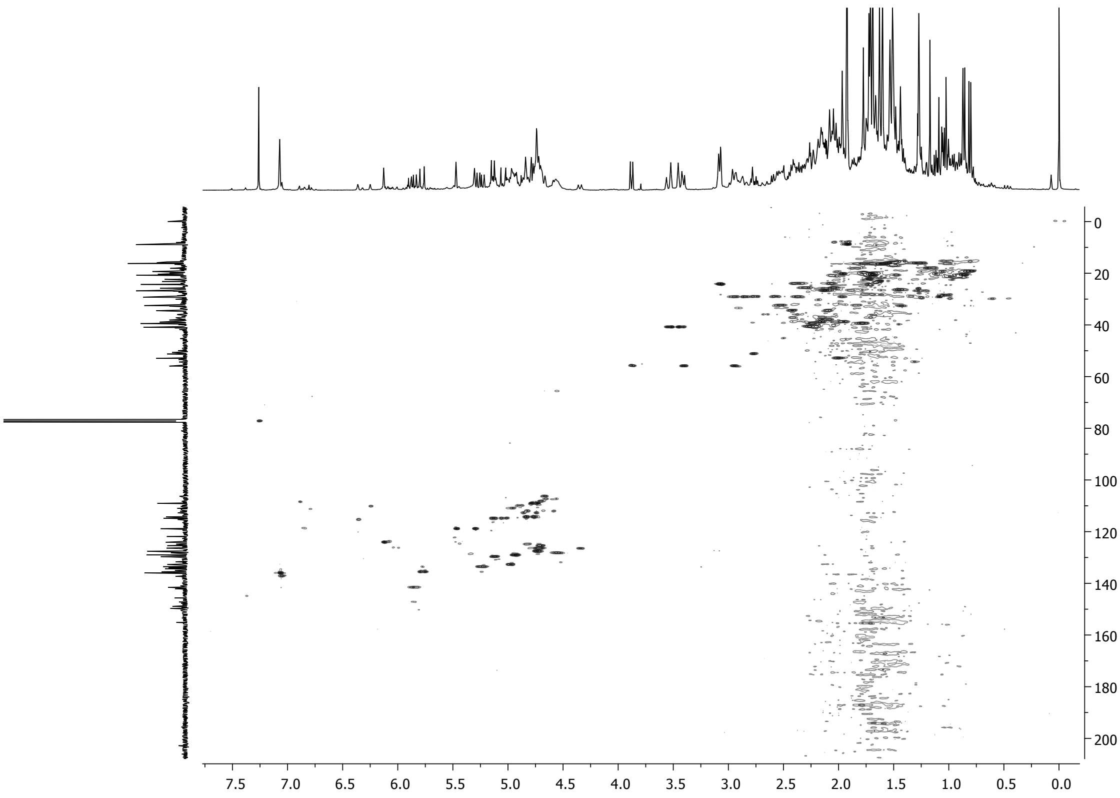

Figure S 4: ^{1}H-^{13}C HSQC of the raw essential oil.

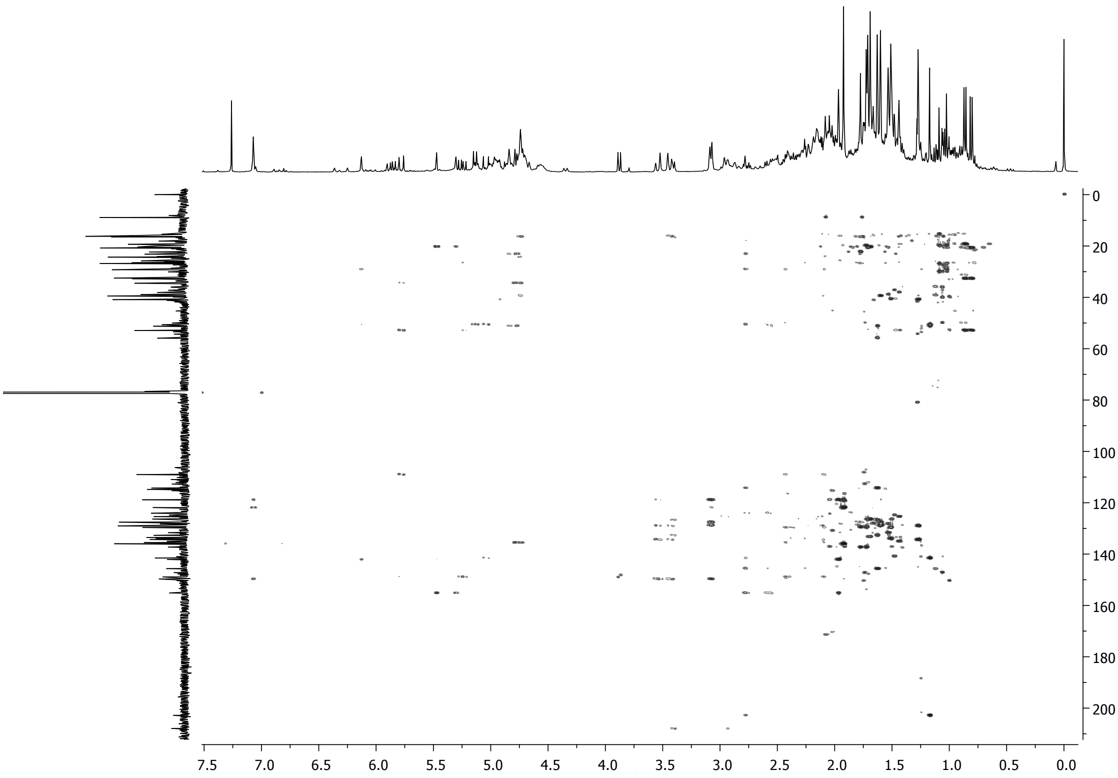

Figure S 5: ^{1}H-^{13}C HMBC of the raw essential oil.

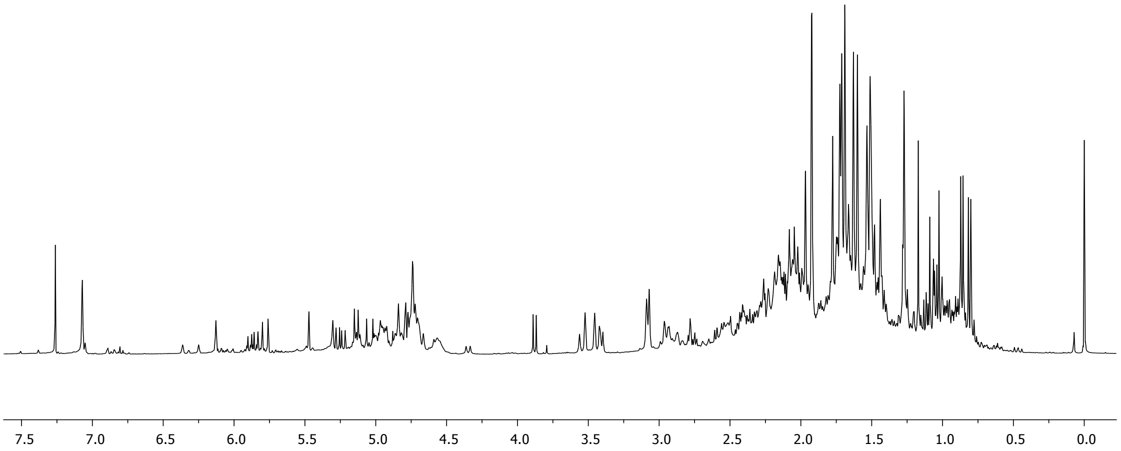

Figure S 6: ^{1}H NMR (400.13 Mhz, $CDCl_3$) spectra of the 60ºC essential oil.

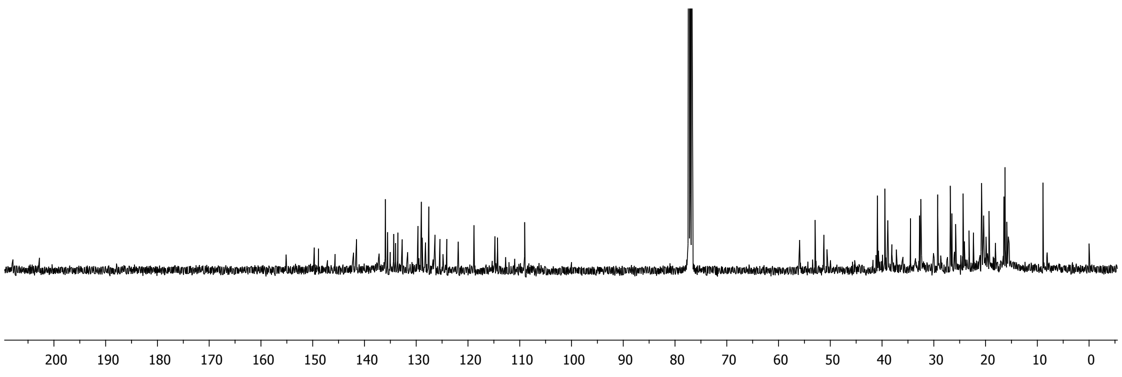

Figure S 7: ^{13}C NMR (100.13 Mhz, $CDCl_3$) spectra of the 60ºC essential oil.

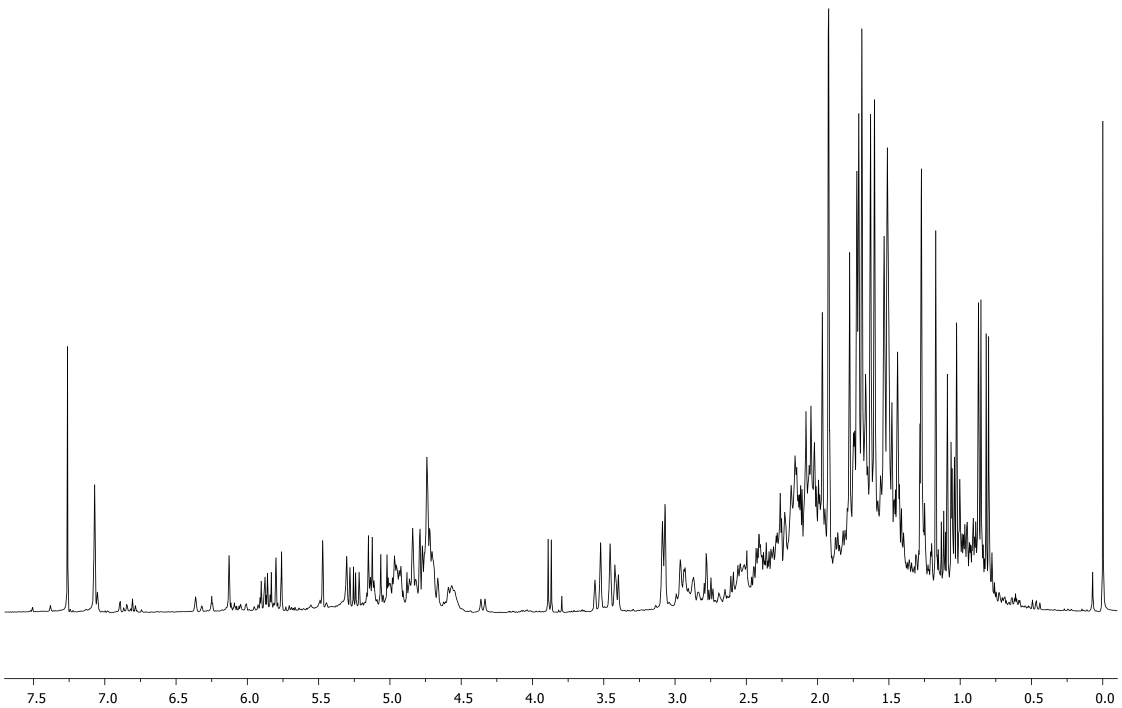

Figure S 8: ^{1}H NMR (400.13 Mhz, $CDCl_3$) spectra of the 120ºC essential oil.

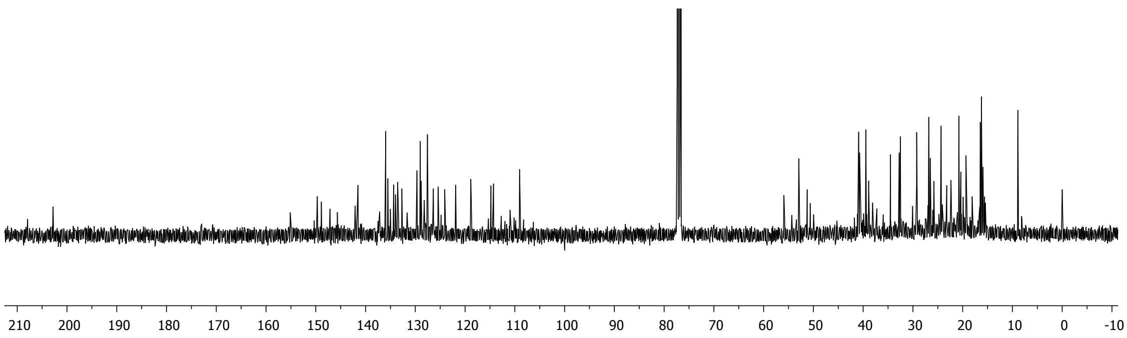

Figure S 9: ^{13}C NMR (100.13 Mhz, $CDCl_3$) spectra of the 120ºC essential oil.

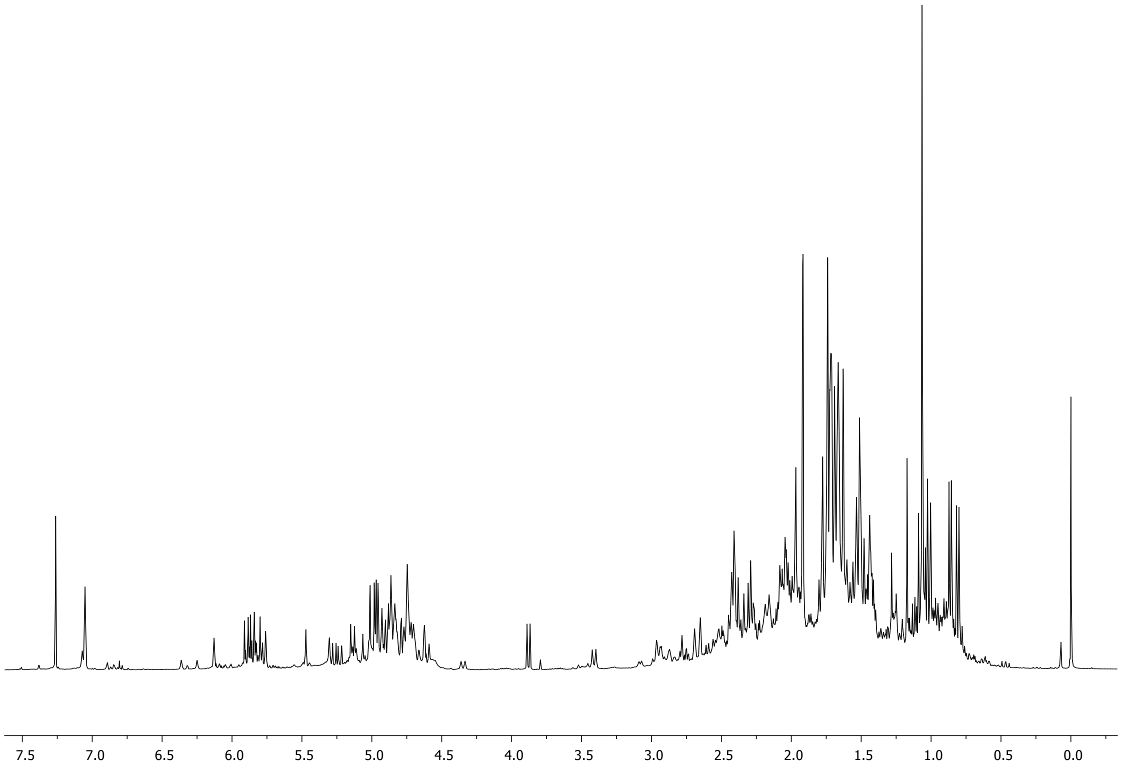

Figure S 10: ^{1}H NMR (400.13 Mhz, $CDCl_3$) spectra of the 180ºC essential oil.

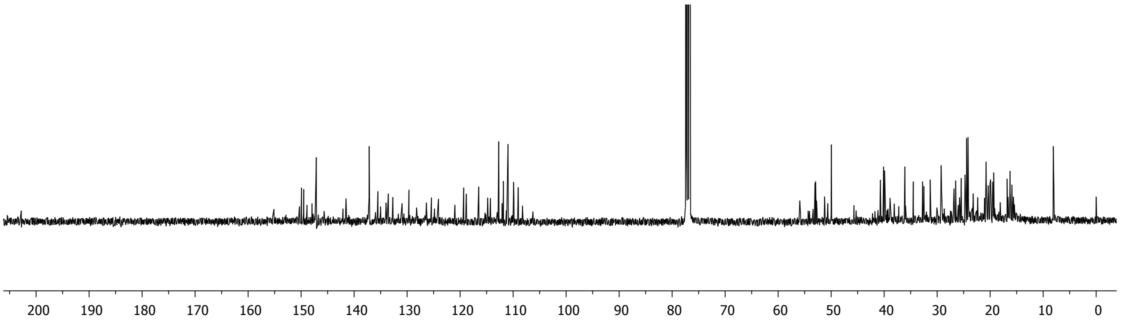

Figure S 11: ^{13}C NMR (100.13 Mhz, $CDCl_3$) spectra of the 180ºC essential oil.

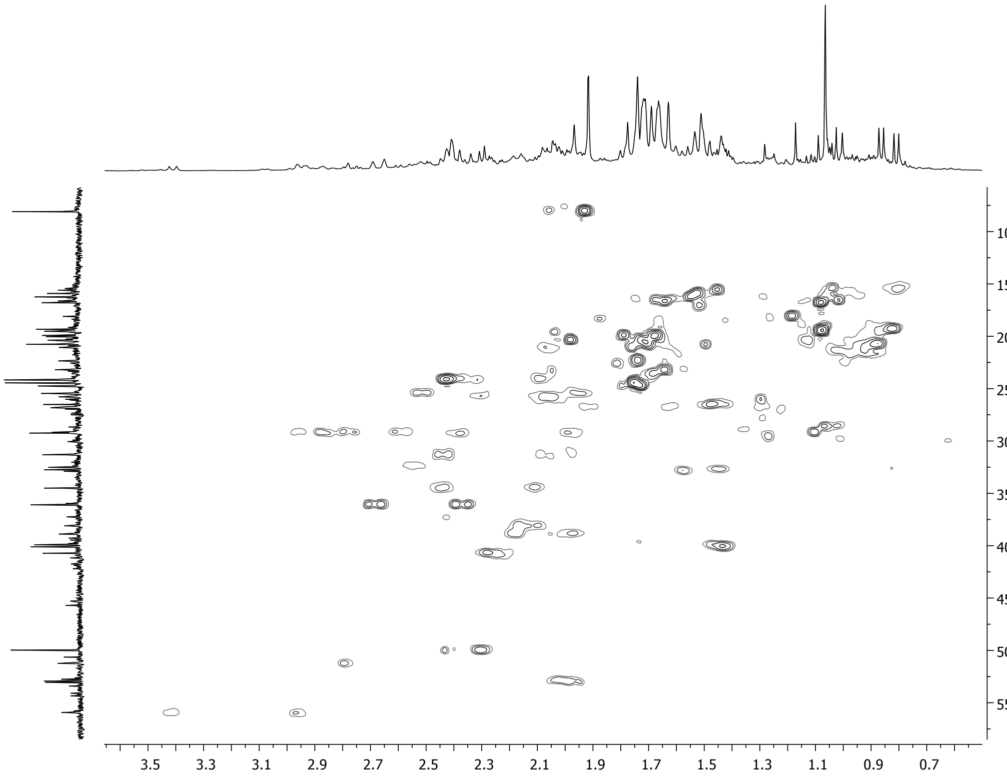

Figure S 12: Selective ^{1}H-^{13}C HSQC of the 180ºC essential oil.

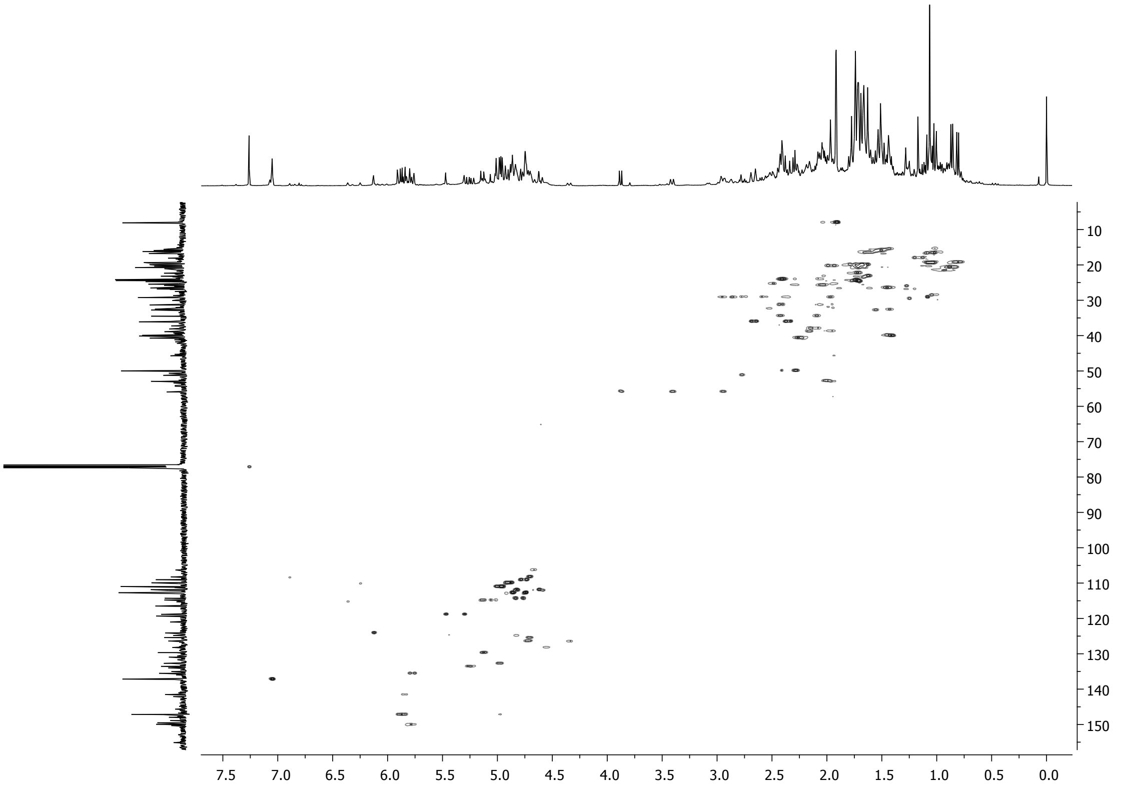

Figure S 13: ^{1}H-^{13}C HSQC of the 180ºC essential oil.

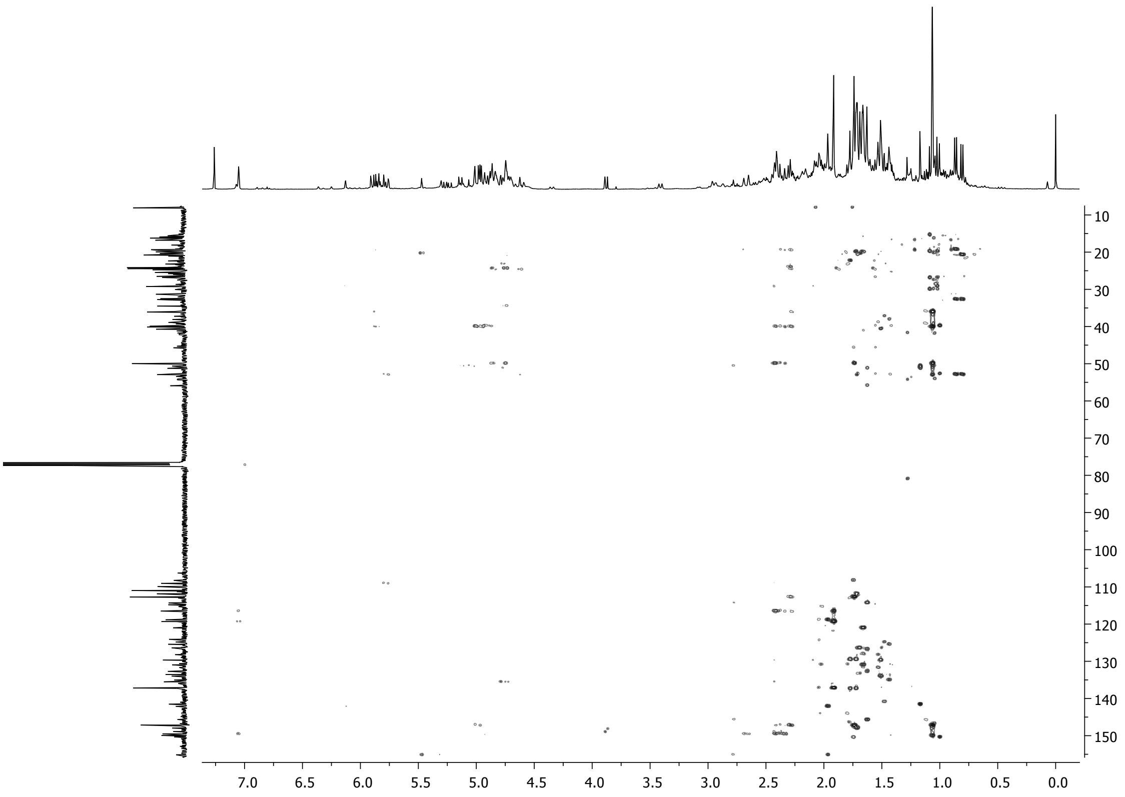

Figure S 14: ^{1}H-^{13}C HMBC of the 180ºC essential oil.

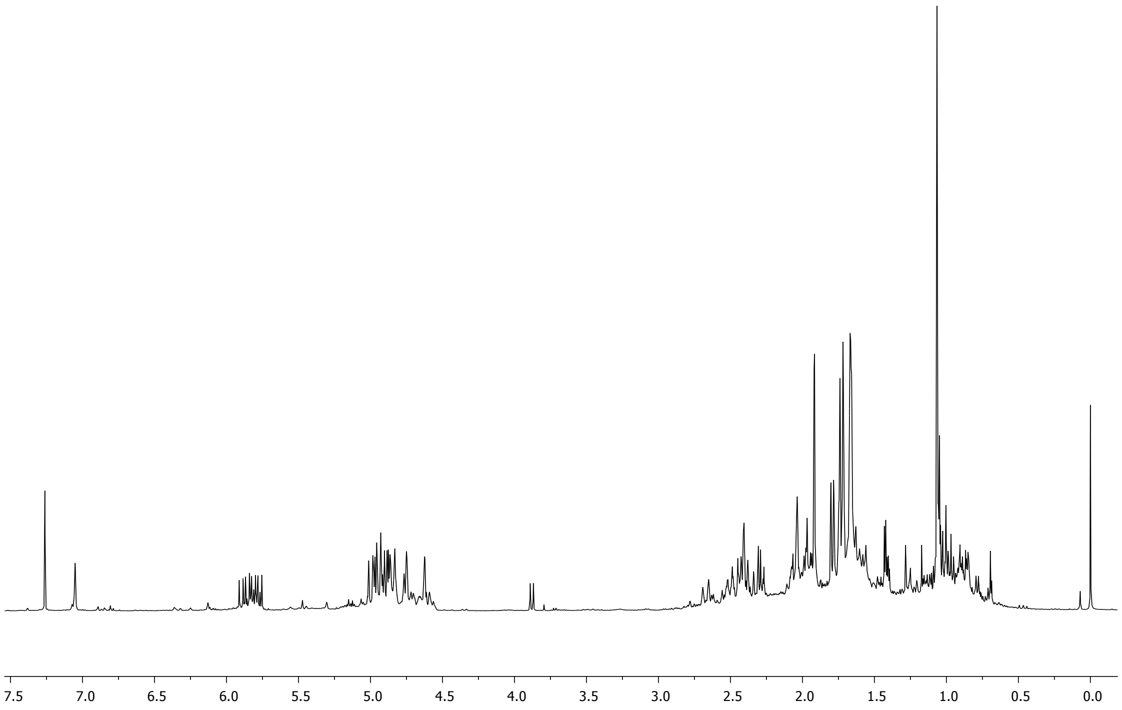

Figure S 15: ^{1}H NMR (400.13 Mhz, $CDCl_3$) spectra of the 240ºC essential oil.

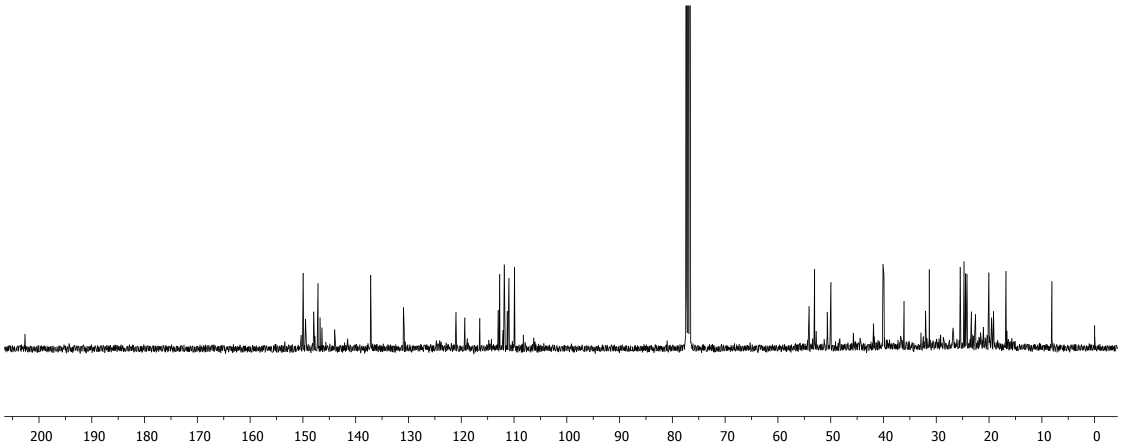

Figure S 16: ^{13}C NMR (100.13 Mhz, $CDCl_3$) spectra of the 240ºC essential oil.

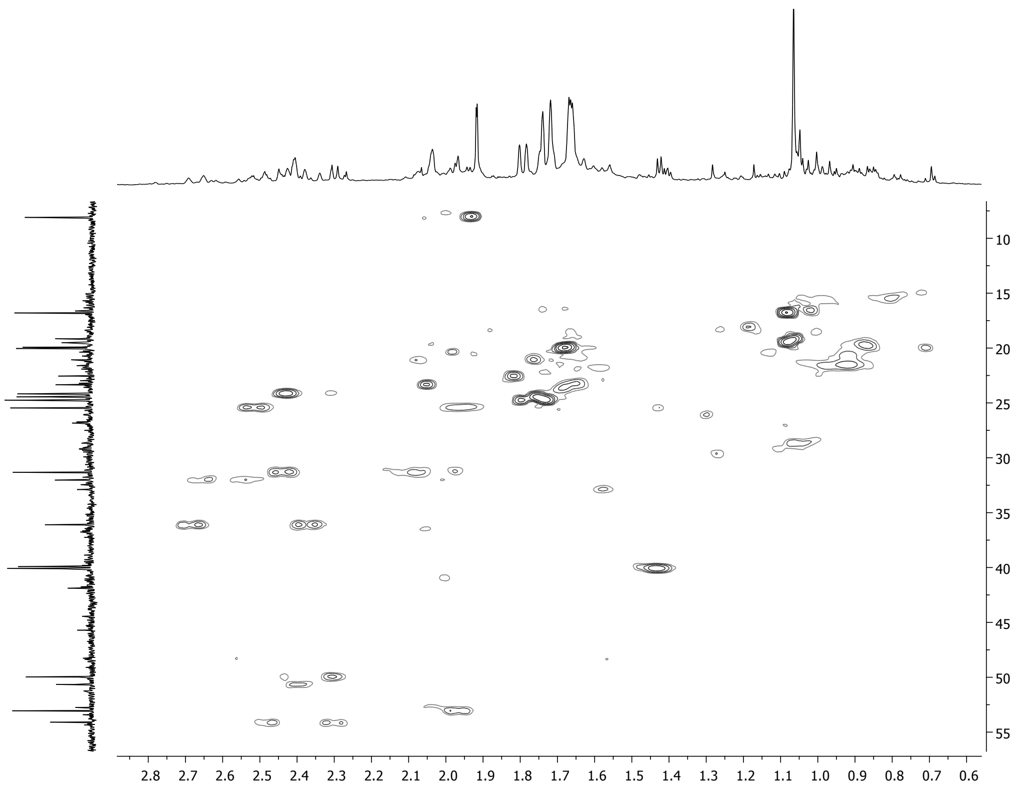

Figure S 17: Selective ^{1}H-^{13}C HSQC of the 240ºC essential oil.

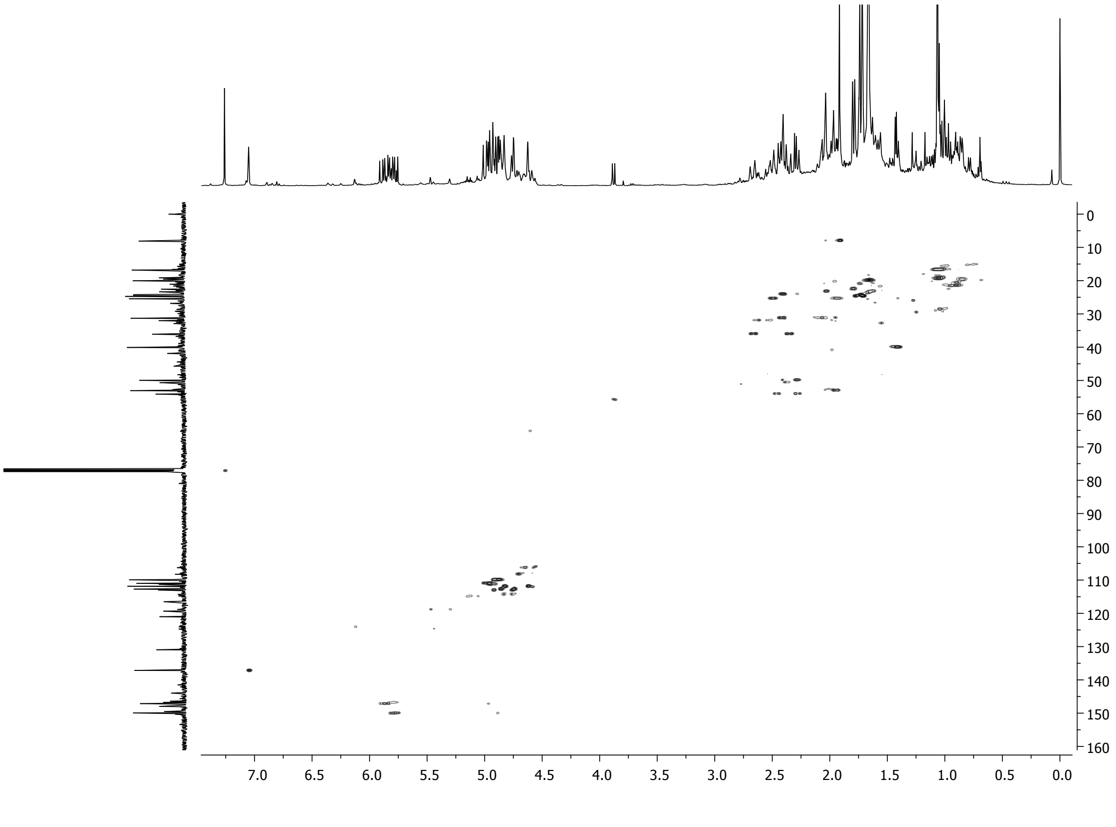

Figure S 18: ^{1}H-^{13}C HSQC of the 240ºC essential oil.

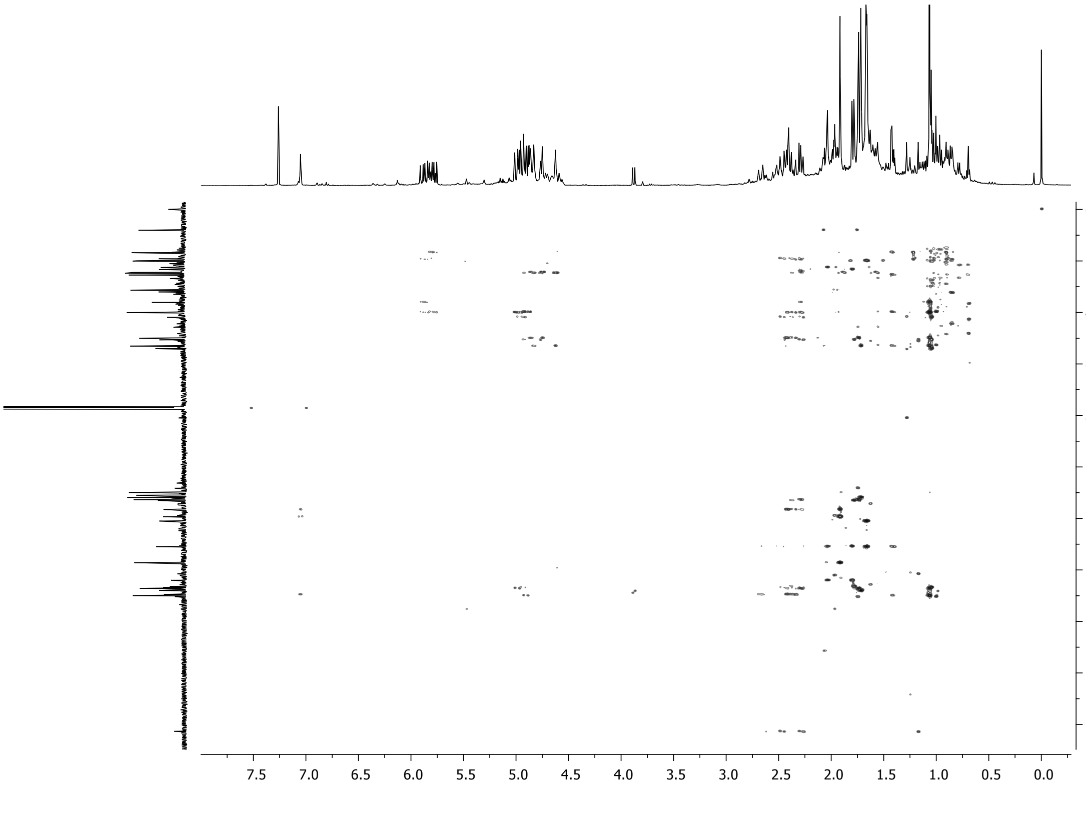

Figure S 19: ^{1}H-^{13}C HMBC of the 240ºC essential oil.

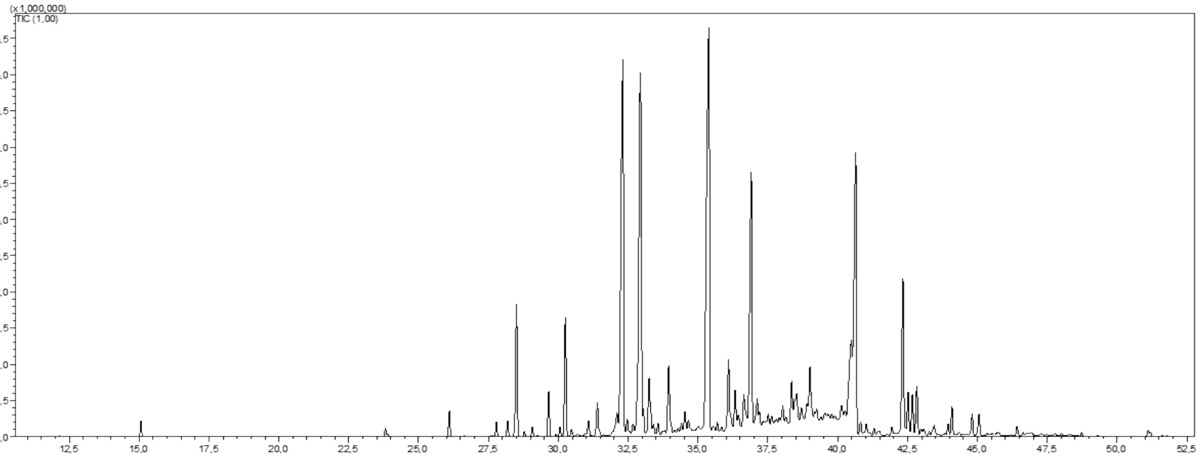

Figure S 20: GC-MS chromatogram of the *Eugenia uniflora* L. essential oil.

Table S1: GC-MS analysis of *E. uniflora* essential oil.

**RRI[a]**	**RRI[b]**	**Compounds**	**Relative area (%)**
1102	1098	Linalool	0,29
1289	1284	Anethole	0,13
1340	1340	δ-Elemene	0,51
1379	1377	α-Copaene	0,28
1388	1384	β-Bourbonene	0,32
1395	1394	β-Elemene	**3,05**
1409	-	n.i	0,18
1423	1418	Caryophyllene	1
1433	-	n.i	0,21
1438	1435	γ-Elemene	**2,67**
1443	1440	Aromandendrene	0,16
1458	1455	α-Humulene	0,3
1466	1457	Alloaromadendrene	0,87
1483	-	n.i	0,46
1488	1480	Germacrene D	**12,64**
1491	-	n.i	0,24
1497	-	n.i	0,19
1503	1500	Curzerene	**13,28**
1506	1499	α-Muurolene	0,15
1511	1510	Bulnesene	1,62
1515	-	n.i	0,18
1519	1524	δ-Cadinene	0,22

1529	1519	Cubebol	1,78
1541	-	n.i	0,15
1544	-	n.i	0,46
1547	1545	Selina-3,7(11)-diene	0,27
1566	1561	Germacrene B	**16,19**
1573	1580	(-)-Globulol	0,17
1584	1582	Spathulenol	1,81
1590	1593	Viridiflorol	0,93
1593	-	n.i	0,31
1598	-	n.i	1,05
1605	1605	β-Elemenone	7,68
1611	-	n.i	0,79
1613	-	n.i	0,24
1622	-	n.i	0,16
1625	-	n.i	0,15
1636	1630	Isospathulenol	0,31
1644	-	n.i	1,03
1649	1640	τ-Muurolol	1,23
1654	1648	τ-Cadinol	0,41
1659	-	n.i	0,55
1662	1662	Cadin-4-en-10-ol	1,51
1668	1669	Atractylone	0,43
1693	-	n.i	0,55
1702	-	Furanodiene	4,3

1707	1696	Germacrone	**9,03**
1712	-	n.i	0,28
1718	-	n.i	0,24
1725	-	n.i	0,15
1744	-	n.i	0,14
1755	1755	Oxidoselina-1,3,7(11)-trien-8-one	**4**
1760	-	n.i	0,92
1765	-	n.i	0,86
1769	1767	Anthracene	1,07
1801	-	n.i	0,21
1805	-	n.i	0,58
1827	-	n.i	0,49
1834	-	n.i	0,46
1875	-	n.i	0,19

RRI[a]: calculated relative retention indices using the column Rtx-5MS (GC–MS) and the *n*-alkanes series C8–C20; RRI[b]: published relative retention indices for non-polar columns. N.i: not identified.
